# Lanthanide-Driven Electronic and Defect Engineering in Spinel Co_3_O_4_: Unraveling the Structure–Activity Synergy for Bifunctional Oxygen Electrocatalysis

**DOI:** 10.3390/ma19153188

**Published:** 2026-07-26

**Authors:** Tianqi Cao, Hongyu Cui, Junyi Liu, Chuanhui Zhang

**Affiliations:** 1 Institute of Materials for Energy and Environment, College of Materials Science and Engineering, Qingdao University, Qingdao 266071, China; 2 Faculty of Chemical Engineering and Energy Technology, Shanghai Institute of Technology, Shanghai 201418, China

**Keywords:** oxygen reduction reaction, oxygen evolution reaction, spinel Co_3_O_4_, lanthanide doping, oxygen vacancy

## Abstract

**Highlights:**

- Citric acid-assisted sol–gel method is adopted to synthesize a series of lanthanide-doped Co_3_O_4_ catalysts.
- Sm-Co_3_O_4_ delivers the smallest potential gap and dominates the high-efficiency 4-electron ORR pathway.
- Sm doping triggers lattice distortion and abundant oxygen vacancies, elevating the Co^2+^/Co^3+^ ratio to 0.59.
- Lanthanide ion doping can simultaneously tune electronic structure and lattice defects of spinel Co_3_O_4_ to enhance oxygen electrocatalytic activity.
- A structure–activity relationship linking lattice defects, Co valence states and catalytic performance is established.

**Abstract:**

The sluggish kinetics of the oxygen reduction reaction (ORR) and oxygen evolution reaction (OER) at air cathodes severely restrict the practical application of rechargeable zinc–air batteries (ZABs). Herein, equimolar lanthanide-doped spinel Co_3_O_4_ bifunctional electrocatalysts were synthesized via a citric acid-assisted sol–gel method. Among Ce-, Pr-, La-, and Sm-doped catalysts, Sm-Co_3_O_4_ exhibits the optimal electrocatalytic performance with a high ORR half-wave potential of 0.72 V and superior OER activity with an overpotential of 1.65 V at 10 mA cm^−2^, achieving a minimal potential gap ΔE of 0.93 V. Rotating ring-disk electrode (RRDE) measurements and Koutecky–Levich (K–L) analyses confirm the exclusive 4e^−^ ORR pathway. Characterizations reveal that Sm doping modulates the electronic structure and lattice distortion of Co_3_O_4_, raises the Co^2+^/Co^3+^ ratio (0.59) and creates abundant oxygen vacancies, thereby significantly lowering the overpotentials for both ORR and OER. This study provides new insights for designing high-performance spinel-based bifunctional electrocatalysts for ZABs.

## 1. Introduction

The excessive exploitation of fossil fuels has precipitated a dual crisis of energy supply and ecosystem degradation, posing severe constraints on global sustainable development [1,2]. Consequently, the exploration of clean, renewable energy systems and the innovation of efficient energy storage and conversion technologies have emerged as pivotal research frontiers and core objectives of national carbon-neutrality strategies [3]. Among emerging electrochemical energy storage technologies, metal–air batteries (MABs) exhibit theoretical energy densities substantially surpassing those of lithium-ion batteries, coupled with intrinsic safety and cost competitiveness, rendering them one of the most promising candidates [4]. In particular, zinc–air batteries (ZABs) utilize earth-abundant zinc metal as the anode, enabling stable charge/discharge cycling in alkaline electrolytes with remarkable economic viability and safety profiles [5,6]. Additionally, ZABs possess a high theoretical gravimetric energy density (1086 W h kg^−1^) and a flat operating voltage plateau, offering substantial potential for large-scale energy storage and portable power applications. Nevertheless, the research advancement and commercialization of ZABs have been persistently hindered by a critical bottleneck: insufficient oxygen electrocatalytic performance at the air cathode [7]. The charge and discharge processes of ZABs rely on two pivotal oxygen electrocatalytic reactions at the cathode: the ORR during discharge and the OER during charge. Both reactions involve multi-electron transfer with complex mechanistic steps, exhibiting intrinsically sluggish kinetics and substantial overpotentials [8]. Therefore, the development of highly efficient bifunctional catalysts is imperative to accelerate reaction kinetics, mitigate polarization losses, and thereby enhance energy conversion efficiency and cycling durability.

Spinel-type Co_3_O_4_ has attracted considerable attention as a promising catalytic material for ORR and OER owing to its abundance and superior redox properties [9,10]. The catalytic performance of spinel Co_3_O_4_ predominantly originates from the coexistence of Co^2+^ and Co^3+^ within the spinel framework. Specifically, Co^2+^ with moderate electron-donating capability facilitates O_2_ adsorption and activation, thereby improving ORR activity, whereas Co^3+^ has been demonstrated to correlate with OER performance [11]. However, pristine Co_3_O_4_ is often constrained by inadequate electrical conductivity and insufficient active site density, resulting in catalytic performance far below theoretical expectations. Elemental doping represents an effective strategy for modulating the electronic structure and defect chemistry of metal oxides, with lanthanide doping garnering increasing attention in recent years. The distinctive properties of lanthanides stem from their progressively filled 4f orbitals, which are spatially confined within the atom and shielded by fully occupied 5s^2^ and 5p^6^ orbitals, rendering the 4f electrons highly localized and minimally involved in chemical bonding [12]. Beyond the characteristic +3 oxidation state, certain lanthanides can stabilize alternative valence states, including +2 (Sm, Eu, Yb) and +4 (Ce, Pr, Tb). This flexible multivalence renders them excellent media for electron storage and transfer [13]. Furthermore, lanthanide ions typically exhibit relatively large ionic radii, and their incorporation into host metal oxide lattices induces significant lattice distortion, serving as a critical driving force for oxygen vacancy formation [14]. Such oxygen vacancies are essential for adsorbing reaction intermediates and regulating the local electronic environment. On the electronic level, the unique 4f orbital of lanthanide ions generates strong 4f–2p–3d orbital hybridization with Co–O units, which adjusts the d-band center of cobalt active sites to achieve moderate adsorption strength for all oxygen-containing reaction intermediates, avoiding the excessively strong or weak binding that restricts reaction kinetics [15]. Structurally, lanthanide substitution introduces controllable lattice strain and abundant oxygen vacancies, which provide extra unsaturated active sites and accelerate the rapid transfer of electrons and electrolytes [16,17,18]. Meanwhile, the reversible valence conversion of lanthanide ions can dynamically regulate the Co^2+^/Co^3+^ redox balance during cyclic oxygen electrocatalysis, effectively inhibiting structural collapse and active site degradation [19,20]. The above electronic modulation and structural optimization synergistically accelerate the reaction kinetics of both ORR and OER, thereby significantly improving the bifunctional catalytic activity of the lanthanide-doped Co_3_O_4_ electrocatalyst. Moreover, coupled reversible Sm^2+^/Sm^3+^ and Co^2+^/Co^3+^ redox pairs accelerate interfacial electron transport to overcome the poor conductivity of pristine Co_3_O_4_, while the robust integrated RE–Co–O lattice inhibits structural degradation during long cycling in Zn–air batteries for sustained catalytic performance [19]. In studies of La-doped Co_3_O_4_ catalysts, La^3+^ substitution for octahedral-site Co^3+^ has been shown to induce lattice distortion, alter the local chemical environment, and enhance oxygen vacancy concentration, synergistically improving redox performance, oxygen species activity, and reaction intermediate conversion efficiency [21].

In this work, a citric acid-assisted sol–gel method was employed to synthesize spinel-type Co_3_O_4_ catalysts doped with equimolar ratio of Ce, Pr, La, or Sm, respectively. The effects of different lanthanide dopants on the ORR and OER catalytic activities of Co_3_O_4_ were systematically compared, and the catalysts were evaluated as cathode materials for zinc–air batteries. The intrinsic origins of the enhanced catalytic activities were elucidated through comprehensive characterization, investigating the phase composition, morphology, and specific surface area, with particular emphasis on oxygen vacancy content and the Co^2+^/Co^3+^ ratio.

## 2. Materials and Methods

Ce-, Pr-, La-, and Sm-doped Co_3_O_4_ catalysts were separately fabricated via a citric acid-assisted sol–gel method. Briefly, 1.92 mmol Co (NO_3_)_2_·6H_2_O (Aladdin Reagents Co., Ltd., Shanghai, China) and 4 mmol citric acid (Aladdin Reagents Co., Ltd.) were dissolved in 2.5 mL deionized water with the addition of 0.08 mmol Ce (NO_3_)_3_·6H_2_O (Adamas Reagent Co., Ltd., Shanghai, China), Pr (NO_3_)_3_·6H_2_O, (Adamas Reagent Co., Ltd.) La (NO_3_)_3_·6H_2_O (Macklin Biochemical Co., Ltd., Shanghai, China) or Sm (NO_3_)_3_·6H_2_O (Adamas Reagent Co., Ltd.) (nominal molar ratio of M/(M + Co) = 1%), respectively. The mixed solution was magnetically stirred at room temperature for 30 min to obtain a homogeneous precursor solution. Subsequently, the above solution was heated to 80 °C under continuous stirring and kept in an 80 °C water bath until a viscous gel was produced. The as-obtained gel was dried overnight in an oven at 120 °C. The brick-red porous dried product was fully ground into fine powder, which was then transferred to a muffle furnace and calcined at 500 °C for 4 h with a heating rate of 2 °C min^−1^. The final black powders were denoted as M-Co_3_O_4_ (M = Ce, Pr, La, Sm) corresponding to various lanthanide dopants. For comparison, pure undoped Co_3_O_4_ was synthesized using identical procedures except that only 2 mmol Co (NO_3_)_2_·6H_2_O was used, which served as the reference catalyst. Additional details of characterizations and electrocatalytic measurements are provided in the Supplementary Materials.

## 3. Results

### 3.1. Characterization Results

**Figure 1:**
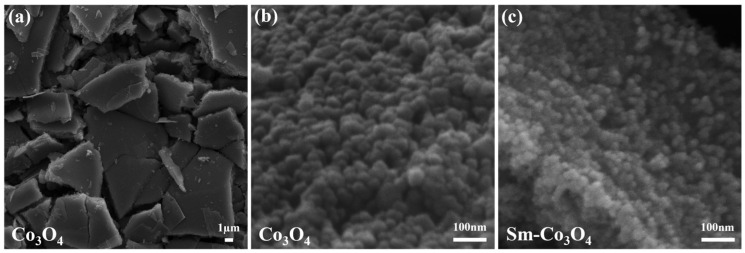
SEM images of (**a**,**b**) Co_3_O_4_ and (**c**) Sm-Co_3_O_4_.

The morphological features of the Co_3_O_4_ and Sm-doped Co_3_O_4_ catalysts were characterized by FE-SEM. As displayed in Figure 1a, pristine Co_3_O_4_ exhibits bulky block-like morphology with smooth surfaces. Such a microstructure facilitates the exposure of highly active crystal facets and the construction of continuous conductive networks, thereby accelerating electron transport. Notably, compared with bare Co_3_O_4_ (Figure 1b), the particle size of Sm-Co_3_O_4_ is remarkably reduced (Figure 1c). Although slight nanoparticle aggregation can be observed visually, the refined primary grains form abundant intergranular mesopores within the aggregates rather than dense stacking. The diminished grain size enlarges the specific surface area, exposes abundant accessible catalytic active sites, and ultimately boosts electrocatalytic performance.

**Figure 2:**
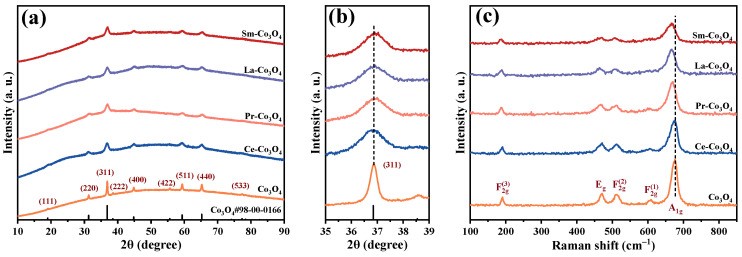
(**a**) XRD patterns of Co_3_O_4_ and M-Co_3_O_4_ catalysts in the 2θ ranges of 10~90° (**b**) and 35~39° and (**c**) Raman spectra of Co_3_O_4_ and M-Co_3_O_4_ catalysts.

Figure 2a displays the XRD patterns of Co_3_O_4_ and M-Co_3_O_4_ series catalysts. All catalysts exhibit consistent characteristic diffraction peaks, which can be unambiguously assigned to the (111), (220), (311), (222), (400), (422), (511), (440) and (533) crystal planes of spinel-type Co_3_O_4_ (JCPDS PDF#98-000-0166). No diffraction peaks corresponding to other metal oxides are detected in M-Co_3_O_4_, revealing that M^n+^ may enter the Co_3_O_4_ lattice, replace cobalt ions within the lattice or occupy interstitial sites, without generating separate impurity oxide phases [22]. Figure 2b presents the magnified view of the (311) characteristic diffraction peak ranging from 35° to 39° 2θ for intuitive comparison. Evidently, all rare-earth doped samples show obvious peak broadening (increased FWHM) and slight peak shift toward higher 2θ angles relative to pure Co_3_O_4_. Among La-, Ce-, Pr- and Sm-doped samples, Sm-Co_3_O_4_ delivers the largest positive offset of the (311) peak. The shift to higher diffraction angles indicates reduced interplanar spacing caused by lattice compressive strain, while peak broadening confirms grain refinement after rare-earth substitution. In accordance with the Scherrer equation, their grain sizes tend to shrink and crystallinity declines, which is consistent with the SEM results.

Raman spectroscopy was employed to characterize the local chemical environment and defect structure of each catalyst, as displayed in Figure 2c. Co_3_O_4_ exhibits five characteristic peaks at approximately 195, 479, 520, 617 and 687 cm^−1^, which are assigned to the $F_{2g}^{\left(1\right)}$, E_g_, $F_{2g}^{\left(2\right)}$, $F_{2g}^{\left(3\right)}$ and A_1g_ vibrational modes of the spinel structure, respectively [23,24]. Among them, the $F_{2g}^{\left(1\right)}$ and E_g_ modes are associated with the bending vibration of O–Co–O at tetrahedral sites (Co^2+^–O^2−^), while the other three modes correspond to the stretching vibration of Co–O at octahedral sites (Co^2+^–O^2−^) [14]. The M-Co_3_O_4_ catalysts also display analogous characteristic peaks corresponding to these five vibrational modes. Combined with the XRD results (Figure 2a), it can be verified that lanthanide metal doping does not disrupt the fundamental spinel structure of Co_3_O_4_. Notably, all characteristic peaks of the M-Co_3_O_4_ catalysts show obvious broadening and red shifts compared with pure Co_3_O_4_. This phenomenon can be attributed to local lattice expansion and elongated bond lengths induced by the incorporation of lanthanide metal ions with larger radii, which weakens Co–O bonds and reduces their force constants [25]. Weakened Co–O bonds facilitate the generation of oxygen vacancies, and abundant oxygen vacancies are regarded as a critical factor for enhancing ORR/OER catalytic activity [26]. Peak broadening indicates an increased density of lattice defects in the catalyst, which favors the formation of more oxygen vacancies and other catalytic active sites [27]. The Sm-Co_3_O_4_ catalyst presents the most prominent red shift and broadening of the A_1g_ peak, signifying that it possesses the highest concentration of lattice defects and the weakest Co–O bonds.

**Figure 3:**
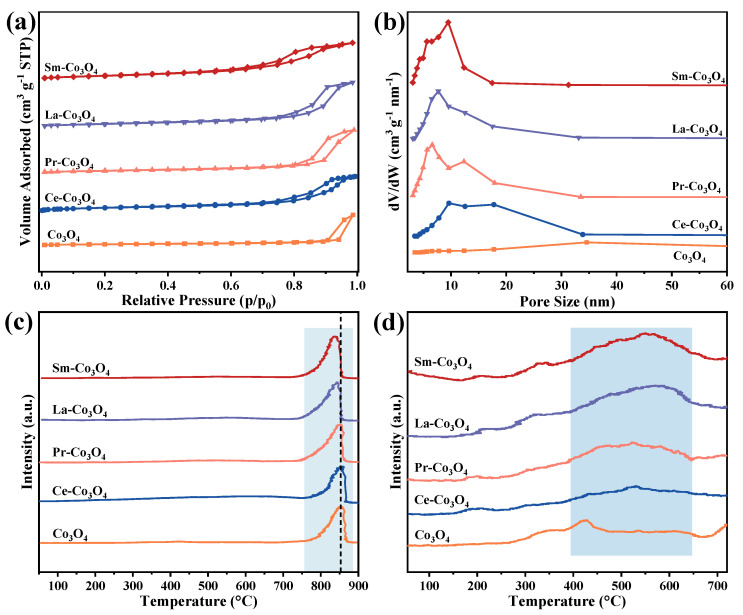
(**a**) N_2_ adsorption–desorption isotherms and (**b**) pore-size distributions of Co_3_O_4_ and M-Co_3_O_4_ catalysts; (**c**) O_2_-TPD profiles of Co_3_O_4_ and M-Co_3_O_4_ and (**d**) enlarged view.

Nitrogen physisorption measurements were conducted on the M-Co_3_O_4_ catalysts to investigate their specific surface area and pore structure characteristics. As shown in Figure 3a, the adsorption–desorption isotherms of all catalysts exhibit Type-IV features with distinct H3 hysteresis loops in the medium-to-high relative pressure (p/p_0_) range, indicating that the catalysts possess typical mesoporous structures dominated by slit pores and intergranular mesopores formed by particle stacking [28]. The closure point of the hysteresis loop for the Sm-Co_3_O_4_ catalyst shifts toward a lower relative pressure, accompanied by a narrower pore size distribution (Figure 3b). This demonstrates that Sm doping optimizes the porous structure of the catalyst and generates mesopores with a narrower pore size [29]. As summarized in Table S1, all rare-earth doping treatments boost the specific surface area and total pore volume of Co_3_O_4_ while reducing average pore size, among which Sm-Co_3_O_4_ displays the optimal porous parameters. Such dramatic porosity modulation of Sm dopant is unique compared with La, Pr and Ce in sol–gel synthesis. Owing to the moderate ionic radius of Sm^3+^, Sm substitution introduces the strongest homogeneous lattice strain to suppress high-temperature grain sintering. Meanwhile, Sm^3+^ forms stable uniform citrate gel precursors, which decompose synchronously during calcination to generate abundant tiny intergranular mesopores. Excessively large La^3+^ and Ce^3+^ cause precursor instability and rare-earth segregation, while Pr^3+^ induces weaker lattice distortion. Benefiting from the above dual effects, Sm-Co_3_O_4_ obtains the maximum specific surface area (67.2 m^2^ g^−1^), highest pore volume (0.19 cm^3^ g^−1^) and minimum pore diameter (10.2 nm), providing richer exposed active sites and faster mass transfer channels for ORR/OER.

The surface oxygen species of M-Co_3_O_4_ catalysts were characterized via O_2_-TPD (Figure 3c). The desorption temperature and peak intensity collectively reflect the type, relative content and binding strength of surface oxygen species on catalysts, and such information is critical for uncovering the origin of catalytic activity toward ORR and OER. The low-temperature desorption peaks (Figure 3d) within the temperature range of 200–400 °C are attributed to weakly adsorbed superoxide species ($O_{2}^{-}$) and peroxide species (O^−^), which are closely associated with ORR activity [30,31]. Notably, the desorption peaks of M-Co_3_O_4_ emerge at lower temperatures in this temperature range, demonstrating that doping endows surface oxygen species with higher intrinsic activity and better activation ability, which facilitates the enhancement of ORR activity. The desorption peaks ranging from 400 to 600 °C correspond to surface lattice oxygen (O^2−^) strongly bound to Co^3+^ sites, and this species is correlated with OER catalytic activity [32,33]. M-Co_3_O_4_ catalysts exhibit intensified desorption peaks and enlarged peak areas in this temperature window, revealing that appropriate lanthanide metal doping can drastically boost the content of surface oxygen species. The intense peak at high temperatures (ca. 800 °C) originates from the desorption of bulk lattice oxygen, a process possibly accompanied by partial decomposition of metal oxides (Figure 3c). It can be observed that M-Co_3_O_4_ catalysts deliver stronger desorption signal with peak positions also shifting toward lower temperatures in this region, which further verifies the weakened Co–O bonds and improved overall reducibility of the catalysts. These findings indicate that the lattice oxygen migration capacity of theses catalysts is enhanced beneficially from the generation of abundant oxygen vacancies [30]. In summary, O_2_-TPD results confirm that M-Co_3_O_4_ delivers favorable conditions for outstanding bifunctional catalytic performance by modulating the content and reactivity of surface oxygen species, among which Sm-Co_3_O_4_ achieves the optimal modification effect.

**Figure 4:**
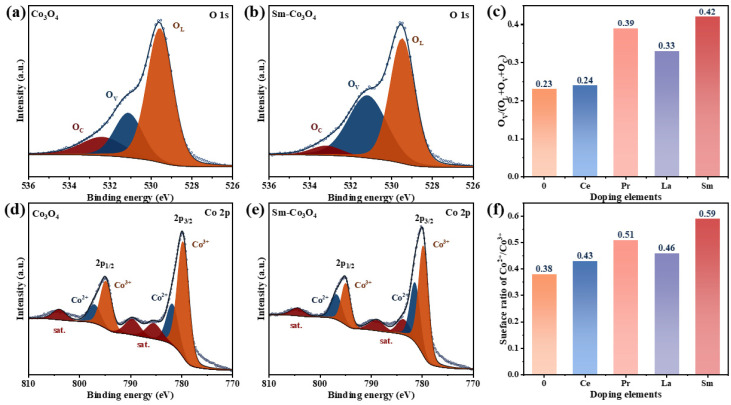
O 1s XPS spectra of (**a**) Co_3_O_4_ and (**b**) Sm-Co_3_O_4_; (**c**) O_V_/O_L_+O_V_+O_C_ ratio; Co 2p XPS spectra of (**d**) Co_3_O_4_ and (**e**) Sm-Co_3_O_4_; (**f**) Co^2+^/Co^3+^ ratio.

The wide-scan XPS spectra in Figure S1 verify that characteristic peaks assignable to Ce, Pr, La and Sm are detected over Ce-Co_3_O_4_, Pr-Co_3_O_4_, La-Co_3_O_4_ and Sm-Co_3_O_4_ apart from C, O and Co signals, respectively, demonstrating the successful incorporation of each lanthanide dopant. Figure 4a,b and Figure S2a–c displayed the deconvoluted O 1s XPS spectra of pristine Co_3_O_4_ and M-doped Co_3_O_4_ catalysts. Three distinct peaks located at 529.2, 530.5 and 531.9 eV can be resolved, which correspond to lattice oxygen (O_L_), oxygen vacancies (O_V_) and chemisorbed oxygen species (O_C_), respectively [34]. Quantitative analysis in Figure 4c reveals that the Sm-Co_3_O_4_ catalyst delivers the highest proportion of surface oxygen vacancies. Moreover, all M-Co_3_O_4_ composites exhibit larger relative oxygen vacancy concentrations than bare Co_3_O_4_. This result indicates that rational lanthanide doping efficiently modulates surface chemical environments and generates abundant active oxygen vacancies and reactive oxygen species, which exert favorable impacts on both ORR and OER. Figure 4d,e and Figure S2d–f present the Co 2p XPS spectra of pristine Co_3_O_4_ and M-doped Co_3_O_4_ catalysts. Two dominant characteristic peaks appear at approximately 779.8 eV and 795.1 eV, which are assigned to the spin–orbit split Co 2p_3/2_ and Co 2p_1/2_ orbitals, respectively [35]. Peak deconvolution identifies two sets of doublets corresponding to Co^2+^ (781.3 and 796.5 eV) and Co^3+^ (779.6 and 794.7 eV), alongside two satellite shake-up peaks [36]. Quantitative analysis in Figure 4f reveals that all lanthanide-doped M-Co_3_O_4_ catalysts possess higher Co^2+^/Co^3+^ ratios than bare Co_3_O_4_. The Co^2+^/Co^3+^ values follow the descending order: Sm-Co_3_O_4_ (0.59) > Pr-Co_3_O_4_ (0.51) > La-Co_3_O_4_ (0.46) > Ce-Co_3_O_4_ (0.43) > Co_3_O_4_ (0.38), which matches the electrocatalytic activity trends. These observations confirm that lanthanide heteroatom doping facilitates the partial reduction of surface Co^3+^ to Co^2+^. Previous literature has documented that elevated Co^2+^ concentration serves multiple beneficial functions for bifunctional oxygen catalysis. Co^2+^ centers act as primary active sites for ORR, facilitating O_2_ adsorption and cleavage of O=O bonds to boost the selectivity of the four-electron ORR pathway. Meanwhile, abundant Co^2+^ also creates a flexible redox environment favorable for OER [37].

**Figure 5:**
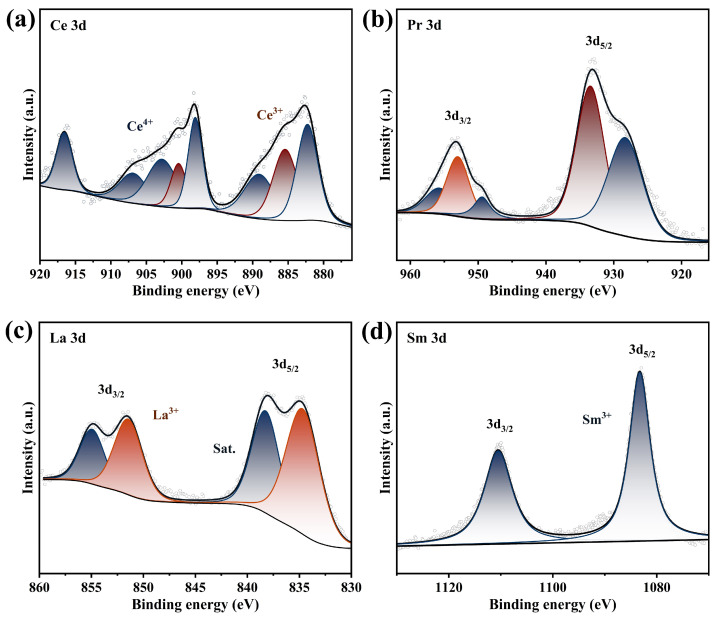
(**a**) Ce 3d XPS spectrum of Ce-Co_3_O_4_; (**b**) Pr 3d XPS spectrum of Pr-Co_3_O_4_; (**c**) La 3d XPS spectrum of La-Co_3_O_4_; and (**d**) Sm 3d XPS spectrum of Sm-Co_3_O_4_.

Figure 5a displays the Ce 3d XPS spectrum of the Ce-Co_3_O_4_ catalyst, which can be divided into two main envelopes assigned to Ce 3d_3/2_ and Ce 3d_5/2_. After peak deconvolution, the two peaks located at 885.4 and 902.8 eV are attributed to Ce^3+^, while the other five peaks correspond to Ce^4+^ [38,39,40]. Figure 5b presents the Pr 3d XPS spectrum of the Pr-Co_3_O_4_ catalyst. Two primary peaks at 933.4 and 953.0 eV originate from Pr 3d_5/2_ and Pr 3d_3/2_, accompanied by their corresponding satellite peaks at 928.3 and 949.4 eV. These results reveal that Pr species coexist in both oxidation states of +3 and +4 [41]. The La 3d XPS spectrum of the La-Co_3_O_4_ catalyst is illustrated in Figure 5c. Two deconvoluted peaks at binding energies of 834.7 and 851.5 eV are characteristic of La^3+^. The peaks at 838.2 and 854.9 eV are assigned to the satellite peaks of La 3d_5/2_ and La 3d_3/2_, respectively [21,42]. Figure 5d shows the Sm 3d XPS spectrum of the Sm-Co_3_O_4_ catalyst, where two dominant peaks emerge at 1083.3 and 1110.5 eV, corresponding to Sm 3d_5/2_ and Sm 3d_3/2_ [19,43,44]. The Sm 3d_5/2_ signal is centered at approximately 1083.3 eV, with a spin–orbit splitting energy of ΔE = 27.2 eV. The broad and asymmetric profiles of these two peaks verify the presence of Sm^3+^ [45,46,47]. The radii of lanthanide M^n+^ ions in M-Co_3_O_4_ catalysts are all larger than those of Co^2+^ and Co^3+^. Benefiting from moderate ionic size matching, M^n+^ cations readily substitute for tetrahedral Co^2+^ sites within the Co_3_O_4_ lattice. The incorporation of large-radius dopant ions into the crystal lattice induces local lattice strain, which can be relieved via the generation of abundant oxygen vacancies [48]. Meanwhile, doping with high-valence metal cations introduces positive charge defects. To maintain overall electrical neutrality of the material system, partial reduction of Co^3+^ to lower-valence Co^2+^ is triggered [49]. This synergistic process elevates the relative Co^2+^/Co^3+^ ratio and simultaneously creates a higher concentration of oxygen vacancies [50].

Collectively, lanthanide heteroatom doping endows Co_3_O_4_ with synergistic electronic and spin modulation effects verified by systematic characterizations and previous theoretical reports. On the electronic level, 4f–2p–3d orbital hybridization redistributes surface charge, elevates the Co^2+^/Co^3+^ ratio and tunes the d-band center for moderate oxygen intermediate adsorption; lattice strain weakens Co–O bonds and generates abundant oxygen vacancies, simultaneously improving intrinsic conductivity and active site density. In terms of spin regulation, lanthanide-induced lattice distortion breaks octahedral crystal field symmetry and triggers spin polarization of Co 3d orbitals to lower the energy barriers of ORR/OER rate-determining steps. Among La, Ce, Pr and Sm dopants, Sm^3+^ delivers the strongest orbital coupling and spin polarization due to the optimal degree of ionic radius mismatch, which provides structural and electronic theoretical foundations for the subsequent electrochemical analysis of various rare-earth modified catalysts.

### 3.2. Electrocatalytic Performance for ORR and OER

**Figure 6:**
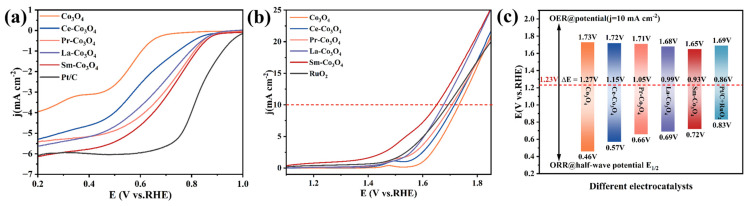
(**a**) LSV curves of ORR over Co_3_O_4_ and M-Co_3_O_4_ in oxygen-saturated electrolyte at 1600 rpm; (**b**) OER polarization curves of Co_3_O_4_ and M-Co_3_O_4_; (**c**) potential difference (ΔE).

The ORR and OER electrocatalytic performances of all catalysts are displayed in Figure 1. Figure 6a shows the ORR polarization curves of pristine Co_3_O_4_ and M-Co_3_O_4_ catalysts. Among all, Sm-Co_3_O_4_ delivers the highest half-wave potential, while Ce-, Pr-, and La-doped counterparts also exhibit markedly elevated half-wave potentials compared with undoped Co_3_O_4_. Figure 6b presents the LSV curves recorded during the OER process. At a current density of 10 mA cm^−2^, Sm-Co_3_O_4_ possesses the lowest overpotential, and all doped catalysts outperform pure Co_3_O_4_. These results reveal that appropriate lanthanide doping can synergistically optimize both ORR and OER catalytic activity of Co_3_O_4_, while Sm doping exhibits the optimal modification effect. To intuitively evaluate the bifunctional electrocatalytic activity, the potential gap ΔE (ΔE = E_10_ − E_1/2_) was calculated, where E_10_ refers to the OER potential at 10 mA cm^−2^ and E_1/2_ represents the ORR half-wave potential. The corresponding histogram of ΔE values is plotted in Figure 6c. A smaller ΔE signifies superior bifunctional catalytic performance. Sm-Co_3_O_4_ simultaneously achieves the maximum E_1/2_ of 0.72 V, the minimum E_10_ of 1.65 V, and the smallest ΔE of 0.93 V, directly verifying its outstanding bifunctional ORR/OER electrocatalytic activity.

**Figure 7:**
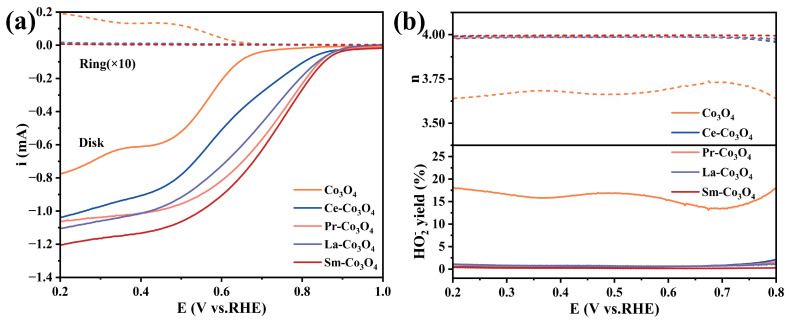
(**a**) RRDE curves and (**b**) electron transfer number (n) and the relative concentration of peroxides intermediate (${HO}_{2}^{-}$) over the Co_3_O_4_ and M-Co_3_O_4_ catalysts.

To uncover the origin of enhanced ORR activity over lanthanide-doped catalysts, rotating ring-disk electrode (RRDE) measurements were carried out for all catalysts. As displayed in Figure 7a, the ring current for ${HO}_{2}^{-}$ oxidation is markedly lower than the disk current, revealing that the ORR preferentially proceeds via an efficient 4e^−^ pathway to produce OH^−^ rather than a 2e^−^ pathway generating ${HO}_{2}^{-}$ [51]. As illustrated in Figure 7b, all M-Co_3_O_4_ catalysts deliver electron transfer numbers close to 4.0 together with extremely low ${HO}_{2}^{-}$ yields, substantially outperforming pristine Co_3_O_4_. This demonstrates that appropriate lanthanide doping greatly boosts the catalyst selectivity toward the desirable 4e^−^ ORR pathway. The previous literature has verified that the 4e^−^ pathway possesses superior energy efficiency and faster reaction kinetics [52,53]. Additionally, this pathway avoids the accumulation of detrimental ${HO}_{2}^{-}$ intermediates, which further facilitates the ORR process.

**Figure 8:**
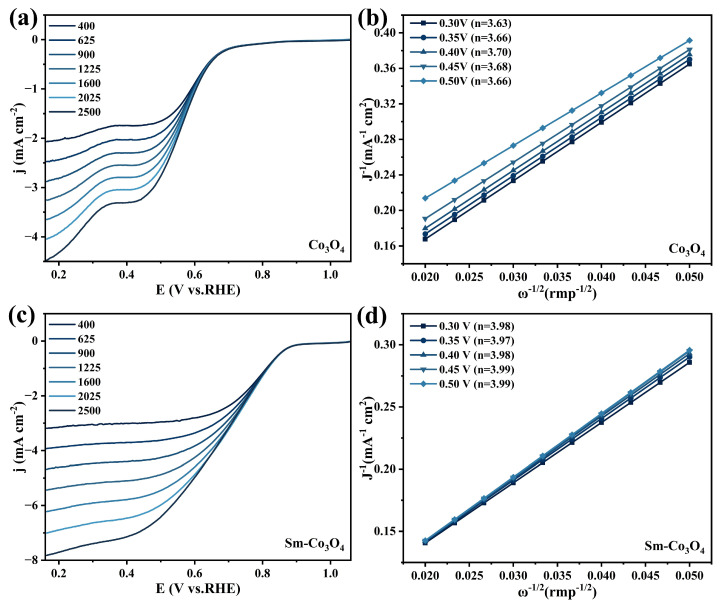
LSV curves of (**a**) Co_3_O_4_ and (**c**) Sm-Co_3_O_4_ in oxygen-saturated 0.1 mol L^−1^ KOH at different rotation rates, K-L plots of (**b**) Co_3_O_4_ and (**d**) Sm-Co_3_O_4_ at different potentials ranging from 0.30 to 0.50 V vs. RHE.

Figure 8a,c display the LSV curves of Co_3_O_4_ and Sm-Co_3_O_4_ recorded at various RDE rotation speeds, respectively. The limiting diffusion current density rises with the increasing rotation speed. This phenomenon originates from the shortened oxygen diffusion distance for ORR at higher rotation rates, which accelerates the mass transport. As presented in Figure 8b,d, the Koutecky–Levich (K–L) plots at different potentials exhibit well-defined linear and nearly parallel features, confirming first-order reaction kinetics with O_2_ diffusion as the mass-transfer-limited step [36,54]. Calculated from the classic K-L equation, the average electron transfer number of Sm-Co_3_O_4_ reaches 3.98, higher than that of Co_3_O_4_ (3.67), which matches the RRDE results.

**Figure 9:**
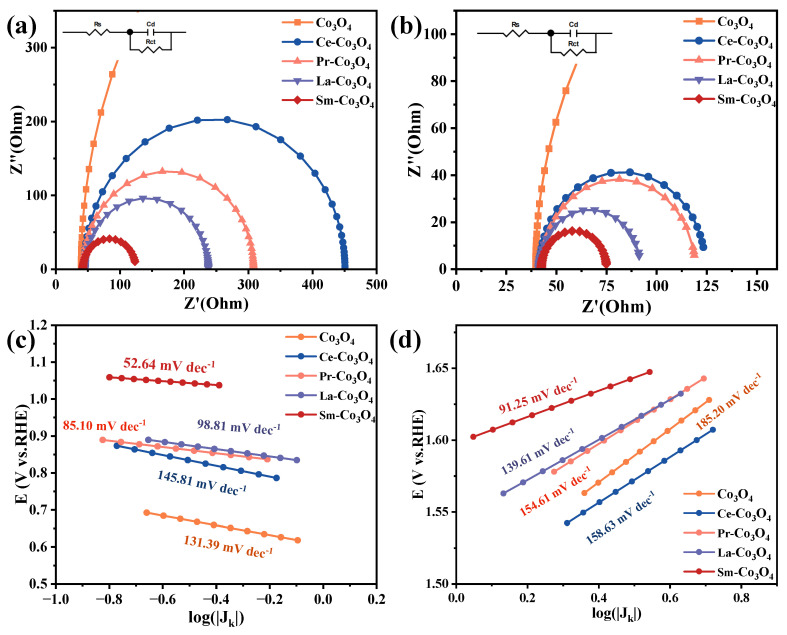
EIS spectra of (**a**) ORR and (**b**) OER over Co_3_O_4_ and M-Co_3_O_4_; Tafel plots derived from the LSV curves of (**c**) ORR and (**d**) OER over Co_3_O_4_ and M-Co_3_O_4_.

**Figure 10:**
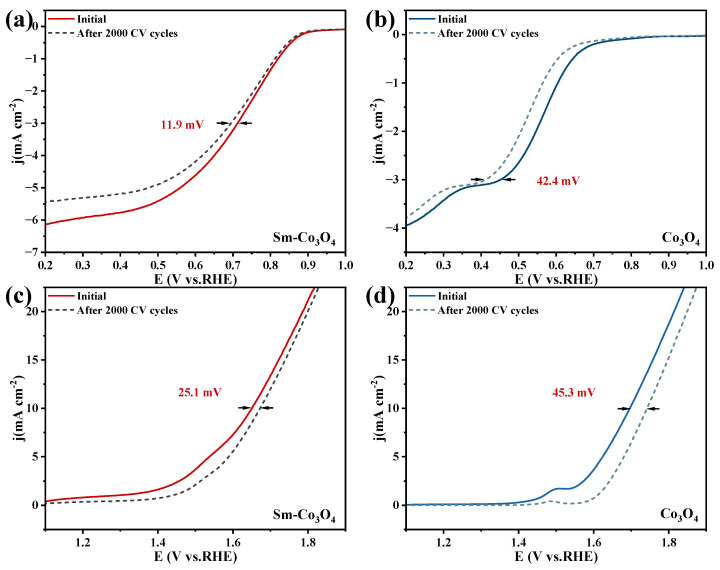
ORR-LSV curves of (**a**) Sm-Co_3_O_4_ and (**b**) Co_3_O_4_ before and after 2000 CV cycles, OER-LSV curves of (**c**) Sm-Co_3_O_4_ and (**d**) Co_3_O_4_ before and after 2000 CV cycles.

Charge transfer resistance (R_ct_) was acquired via Electrochemical impedance spectroscopy (EIS) measurements. As shown in Figure 9a,b, Sm-Co_3_O_4_ delivers the smallest R_ct_ values for both ORR and OER, which are 82.44 Ω and 32.59 Ω, respectively. This reveals its favorable electrical conductivity and rapid faradaic reaction kinetics, thereby endowing superior electrocatalytic activity. The outstanding conductivity of Sm-Co_3_O_4_ accelerates electron transfer across the electrode–electrolyte interface. In addition, the Tafel slope serves as a critical indicator to evaluate ORR and OER kinetics. As depicted in Figure 9c,d, Sm-Co_3_O_4_ exhibits much lower Tafel slopes (52.64 mV dec^−1^ for ORR, 91.25 mV dec^−1^ for OER) in comparison with pristine Co_3_O_4_ (131.39 mV dec^−1^ for ORR, 185.20 mV dec^−1^ for OER). A smaller Tafel slope corresponds to faster reaction kinetics, demonstrating that Sm-Co_3_O_4_ possesses the optimal catalytic efficiency toward both ORR and OER. The ORR stability of the catalyst was assessed by comparing the LSV curves acquired before and after 2000 CV cycles (Figure 10a,b). These results reveal that the Sm-Co_3_O_4_ catalyst exhibits a negligible negative shift in E_1/2_ of only ca. 11.9 mV, which is significantly smaller than Co_3_O_4_ (42.4 mV). In terms of OER performance, the overpotential of Sm-Co_3_O_4_ only positively shifts by 25.1 mV after 2000 CV cycles (Figure 10c), while pure Co_3_O_4_ suffers a much larger positive shift of 45.3 mV (Figure 10d). These contrastive results fully demonstrate the superior bifunctional cycling durability of Sm-doped Co_3_O_4_. Collectively, the ORR and OER results demonstrate that the Sm-Co_3_O_4_ catalyst exhibits an excellent bifunctionally electrocatalytic activity, outperforming the pristine Co_3_O_4_.

## 4. Discussion

In this study, equimolar Ce-, Pr-, La-, and Sm-doped spinel Co_3_O_4_ bifunctional oxygen electrocatalysts were separately fabricated via a citric acid-assisted sol–gel route. Electrochemical measurements demonstrate that lanthanide metal doping synchronously enhances the ORR and OER catalytic activities of the bare Co_3_O_4_, with Sm-Co_3_O_4_ delivering the optimal overall performance. Sm doping strengthens the selectivity of the four-electron ORR pathway, accelerates interfacial electron transport, and optimizes the kinetics of oxygen electrocatalysis. Physicochemical characterizations reveal the structure–activity mechanism regulated by doping, where Sm doping refines grain size and constructs uniform mesoporous structures to enrich exposed active sites. The lattice substitution of large-radius lanthanide ions generates lattice strain, weakens Co–O bonds and increases defect density, thus inducing abundant oxygen vacancies that act as intrinsic active sites for ORR and OER. Meanwhile, lattice positive charge defects promote the conversion of Co^3+^ to Co^2+^ and raise the surface Co^2+^/Co^3+^ ratio. This heteroatom doping strategy enables precise regulation of lattice defects, oxygen vacancy concentration and Co valence distribution in spinel Co_3_O_4_, offering a universal design guideline for developing high-performance spinel-based bifunctional oxygen electrocatalysts.

## Data Availability

The original contributions presented in this study are included in the article/Supplementary Material. Further inquiries can be directed to the corresponding author.

## Supplementary Materials

The following supporting information can be downloaded at https://www.mdpi.com/article/10.3390/ma19153188/s1. Figure S1: Wide-scan XPS spectra of Co_3_O_4_ and M-Co_3_O_4_; Figure S2: O 1s XPS spectra of (a) Ce-Co_3_O_4_, (b) Pr-Co_3_O_4_ and (c) La-Co_3_O_4_, Co 2p XPS spectra of (d) Ce-Co_3_O_4_, (e) Pr-Co_3_O_4_ and (f) La-Co_3_O_4_; Table S1: Textural properties of the catalyst derived from the nitrogen physisorption measurements.
