# Supplementary material for: Lanthanide-Driven Electronic and Defect Engineering in Spinel Co3O4: Unraveling the Structure–Activity Synergy for Bifunctional Oxygen Electrocatalysis
Source: Materials (Basel). 2026 Jul 26;19(15):3188. doi: 10.3390/ma19153188 (PMC13466421; doi:10.3390/ma19153188)
Supplement: Supplementary file 1 [file materials-19-03188-s001.zip › materials-4438046-supplementary.pdf]

Lanthanide-Driven Electronic and Defect Engineering in Spinel Co_3O_4: Unraveling the Structure-Activity Synergy for Bifunctional Oxygen Electrocatalysis

Tianqi Cao [1], Hongyu Cui [1], Junyi Liu [1], Chuanhui Zhang [1, 2, *]

[1] Institute of Materials for Energy and Environment, College of Materials Science and Engineering, Qingdao University, Qingdao 266071, China
[2] Faculty of Chemical Engineering and Energy Technology, Shanghai Institute of Technology, Shanghai 201418, China
* Correspondence: zhangch@qdu.edu.cn

1. Catalyst characterization

Field-emission scanning electron microscopy (FE-SEM, JEOL JSM-7800F) was used to characterize the morphology features of the catalysts. X-ray diffraction (XRD) patterns were collected on an X-ray diffractometer (Rigaku Ultima IV, 40 kV) with Cu-Kα radiation (λ = 0.15418 nm). Nitrogen adsorption-desorption isotherms were measured on a Quantachrome Autosorb-IQ3 automated sorption system. The specific surface areas were computed using the Brunauer-Emmett-Teller (BET) method. Raman spectra were recorded on a Renishaw inVia plus Micro-Raman spectrometer using a 50 mW DPSS laser with 532 nm excitation wavelength. The temperature-programmed desorption of oxygen (O_2-TPD) technique was employed to conducted on a chemisorption analyzer (Quantachrome, Chembet Pulsar). A sample was pretreated under the conditions of 3 vol.% O_2 in Helium at 300 °C for 2 h. Then, the sample was cooled to room temperature followed by heating to 900 °C at a ramp of 10 °C min^{-1} in He. The X-ray photoelectron spectroscopy (XPS) technique was used to analyze the chemical composition and metal valances with the C 1s binding energy (284.6 eV) of adventitious carbon as an internal standard to calibrate all spectra on a PHI 5000 Versa Probe III equipment using Al Kα radiation (1486.6 eV) as the radiation source flow.

2. Electrochemical measurements

Electrochemical measurements of the catalysts were conducted using the three-electrode testing system on an Autolab Potentiostat PGSTAT 302N at room temperature. First, 5 mg of catalyst powder and 2.5 mg of Cabot Vulcan carbon (XC-72) were dispersed into a mixed solution containing 750 μL of deionized water, 250 μL of isopropyl alcohol, and 20 μL of Nafion (5 wt.%). The mixture was subjected to ultrasonic treatment for 30 min to prepare a homogeneous catalyst ink. The working electrode was fabricated by depositing 10 μL of the catalyst ink onto the glassy carbon surface of the rotating ring-disk electrode (RRDE) with an area of 0.19625 cm^{-2}, resulting in a catalyst loading value of 0.25 mg cm^{-2}. The Hg/HgO was used as the reference electrode and platinum foil (1 cm^2) served as the counter electrode. The electrolyte was employed by 0.1 mol L^{-1} KOH solution. For comparison, commercial Pt/C and RuO_2 were also evaluated in ORR and OER under the same testing conditions, respectively. The potential data obtained from the Hg/HgO electrode ($E_{Hg/HgO}$) was converted to potentials vs. the reversible hydrogen electrode (E_{RHE}) using the following formula:

$$E_{RHE} = E_{Hg/HgO} + 0.059 \times \mathrm{pH} + 0.098 \quad \text{(S1)}$$

In an oxygen-saturated KOH electrolyte, high-purity oxygen (99.99%) was continuously bubbled for 30 min. The linear sweep voltammetry (LSV) curves were collected with a scanning speed of 2 mV·s^{-1} under rotating speeds ranging from 400 to 2500 rpm. The transferred electron number (n) was calculated by the Koutecky-Levich (K-L) equations:

$$\frac{1}{J} = \frac{1}{J_K} + \frac{1}{B\omega^{1/2}} \quad \text{(S2)}$$

$$B = 0.2nFD_0^{2/3}v^{-1/6}C_0 \quad \text{(S3)}$$

$$J_K = nFKC_0 \quad \text{(S4)}$$

where J (mA cm^{-2}) is the measured current density, J_L (mA cm^{-2}) and J_K (mA cm^{-2}) are the diffusion- and kinetic- limiting current densities, ω (rpm) is the electrode rotation rate, F is the Faraday constant (96,500 C mol^{-1}), C_0 is the bulk concentration of oxygen (1.2×10^{-6} mol cm^{-3}), D_0 is the diffusion coefficient of oxygen in the electrolyte (1.9×10^{-5} cm^2 s^{-1}), v is the kinematic viscosity of the electrolyte and K is the electron-transfer rate constant.

The rotating ring-disk electrode (RRDE) curves were recorded at a scanning rate of 2 mV s^{-1} in an oxygen-saturated 0.1 M KOH electrolyte. The HO_2^-% and n are determined according to the following formulas:

$$HO_2^-\% = 100\frac{2\, I_r/N}{I_d + I_r/N} \quad \text{(S5)}$$

$$n = 4\frac{NI_d}{NI_d + I_r} \quad \text{(S6)}$$

where I_d (mA) is the disk current, I_r (mA) is the ring current and N = 0.249 is the current collection efficiency of the platinum ring.

Tafel slopes were analyzed by the Tafel equations:

$$\eta = a + b \times \log |\, j \,| \quad \text{(S7)}$$

$$a = \frac{RT}{\alpha F}\log j^0 \quad \text{(S8)}$$

$$b = -\frac{RT}{\alpha F} \quad \text{(S9)}$$

Where η (V) is the overpotential, j^0 (A) is the exchange current density, R is the universal gas constant (8.314 J mol^{-1} K^{-1}), T (K) is the absolute temperature, α is the transfer coefficient, and F is the Faraday constant (96,500 C mol^{-1}).

The LSV curves were acquired at a scanning rate of 2 mV s^{-1} in an oxygen-saturated KOH electrolyte to evaluate the OER performance of the catalysts. The electrochemical impedance spectroscopy (EIS) measurements were conducted at 1.55 V (vs. RHE).

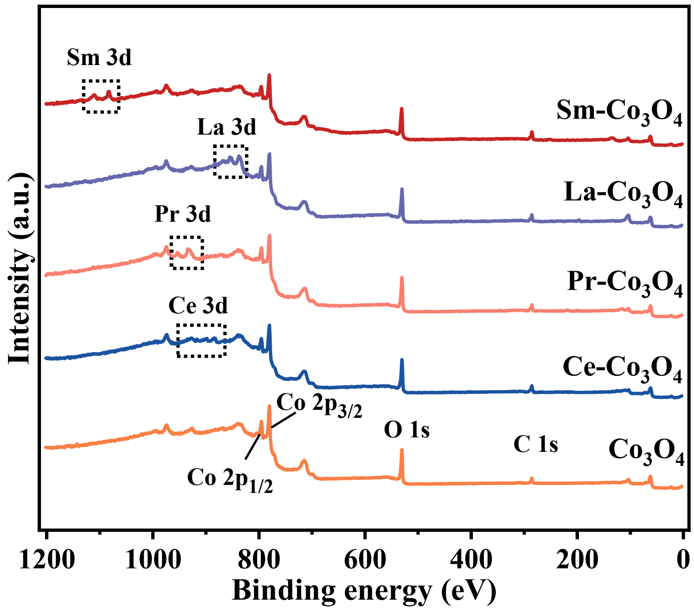

Figure. S1 Wide-scan XPS spectra of Co_3O_4 and M-Co_3O_4

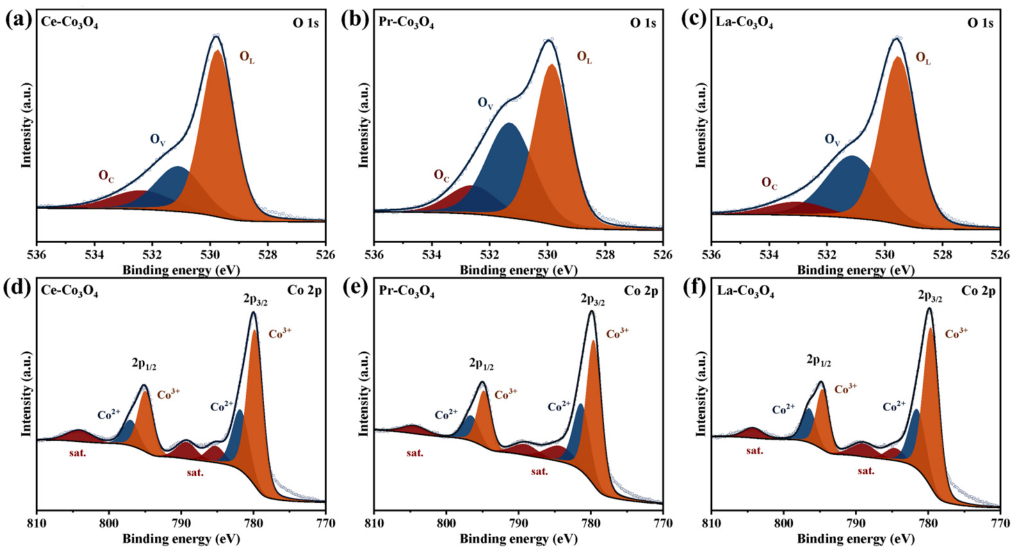

Figure. S2 O 1s XPS spectra of (a) Ce-Co_3O_4, (b) Pr-Co_3O_4 and (c) La-Co_3O_4, Co 2p XPS spectra of (d) Ce-Co_3O_4, (e)Pr-Co_3O_4 and (f)La-Co_3O_4

Table S1 Textural properties of the catalyst derived from the nitrogen physisorption measurements

Catalysts	Specific Surface Area[a] (m^2 g^{-1})	Total Pore Volume[b] (cm^3 g^{-1})	Pore Diameter[c] (nm)
Co_3O_4	19.2	0.12	34.6
Ce-Co_3O_4	32.6	0.16	18.1
Pr-Co_3O_4	58.4	0.17	13.8
La-Co_3O_4	56.9	0.15	12.1
Sm-Co_3O_4	67.2	0.19	10.2

[a] Determined by the Brunauer-Emmett-Teller method.

[b] Estimated from the volume adsorbed at the relative pressure $p/p_0 = 0.99$.

[c] Determined by the Barret-Joyner-Halenda method.
